# Assessing Cognitive Responses and Serum Vitamin B₁₂ Levels in Newly Diagnosed Type 2 Diabetes Mellitus Patients Treated With Metformin: A Prospective Study

**DOI:** 10.7759/cureus.105493

**Published:** 2026-03-19

**Authors:** Santosh Wakode, Rahul Gour, Suryabhan L Lokhande, Abhishek Singhai, Ankur Wakode, Rajay Bharshankar, Jitendra Singh, M. Sukumar Sukumar

**Affiliations:** 1 Physiology, All India Institute of Medical Sciences, Bhopal, Bhopal, IND; 2 Biochemistry, All India Institute of Medical Sciences, Bhopal, Bhopal, IND; 3 General Medicine, All India Institute of Medical Sciences, Bhopal, Bhopal, IND; 4 Anatomy, All India Institute of Medical Sciences, Nagpur, Nagpur, IND; 5 Translation Medicine, All India Institute of Medical Sciences, Bhopal, Bhopal, IND

**Keywords:** cognition, event-related potentials (p300), metformin, type 2 diabetes mellitus, vitamin b₁₂

## Abstract

Background

Type 2 diabetes mellitus (T2DM) is associated with cognitive impairment, and metformin, the first-line pharmacotherapy, has been linked to vitamin B₁₂ deficiency with potential neurological consequences. Objective evidence linking early metformin-induced changes in vitamin B₁₂ levels to cognitive function remains limited, particularly in Indian populations.

Objectives

To evaluate changes in serum vitamin B₁₂ status and cognitive function, assessed using P300 event-related potentials (ERPs), after six months of metformin therapy in newly diagnosed T2DM patients.

Methods

This prospective observational study included 52 adults (18-60 years) with newly diagnosed T2DM who were initiated on metformin monotherapy. Baseline and six-month assessments included serum vitamin B₁₂, homocysteine, methylmalonic acid (MMA), HbA1c, and cognitive evaluation using auditory oddball P300 ERPs. Changes over time were analyzed using the Wilcoxon signed-rank test, and correlations were assessed using Spearman’s coefficient.

Results

After six months, blood sugar control improved significantly. The HbA1c level decreased from 7.8 (6.9-8.9) to 7.2 (6.5-8.1) (p = 0.001). However, serum vitamin B₁₂ levels decreased significantly, from 506.7 (347.3-613.5) to 485.0 (332.4-592.0) (p < 0.001). At the same time, homocysteine and MMA levels increased. Homocysteine increased from 16.5 (10.1-23.9) to 17.2 (10.8-25.0) μmol/L (p < 0.001), suggesting the development of functional vitamin B₁₂ deficiency.

Additionally, P300 latency increased significantly at the frontal, central, and parietal electrode sites (p < 0.001), indicating slower cognitive processing. Although vitamin B₁₂ levels decreased, no statistically significant correlation was found between serum vitamin B₁₂ levels and P300 latency.

Conclusion

In newly diagnosed Indian patients with type 2 diabetes mellitus, six months of metformin therapy improved blood sugar control but was associated with early signs of functional vitamin B₁₂ deficiency and slower cognitive processing, as indicated by increased P300 latency. These results highlight the importance of regular monitoring of vitamin B₁₂ status, including functional markers like homocysteine and MMA. Early detection and correction of vitamin B₁₂ deficiency may help preserve cognitive function and support better long-term outcomes in patients on metformin therapy.

## Introduction

Diabetes, a chronic progressive metabolic disorder of heterogeneous etiopathology characterized by the pathognomonic feature of hyperglycemia in the absence of treatment, is a leading public health concern worldwide [1]. According to the recently published Global Burden of Disease, Injuries, and Risk Factors Study (GBD 2021), the global age-standardized prevalence of diabetes has increased by 90.5% since 1990, resulting in a worldwide burden of 529 million diabetic cases in 2021 [2]. With projections estimating that this number could increase to 1.27 billion by 2050, low- and middle-income countries, particularly India, which reported 78.8 million cases in 2021, bear a disproportionate share of this growing epidemic [3]. The persistent rise in type 2 diabetes mellitus (T2DM) places a significant strain on healthcare systems worldwide, underscoring the urgent need for effective management strategies [4].

Optimal glycemic control remains paramount in minimizing the adverse complications associated with T2DM, including its detrimental effects on the nervous system [5]. The primary intervention for newly diagnosed patients often involves monotherapy with metformin, an oral hypoglycemic agent, combined with lifestyle modifications unless contraindicated. Metformin, a synthetic biguanide, has been the cornerstone of glucose-lowering therapy for over six decades and is currently prescribed to more than 200 million diabetic patients worldwide, either as monotherapy or in combination with other agents [6]. Despite its widespread use, the precise mechanisms underlying metformin’s effects remain poorly understood, with emerging evidence suggesting potential effects on vitamin B12 metabolism [7].

Recent studies have suggested a possible association between long-term metformin use and vitamin B12 deficiency, with significant neurological implications [8]. Meta-analyses compiling data from diverse populations reveal that diabetic patients on metformin therapy are at a higher risk of developing vitamin B12 deficiency, with lower serum levels observed in comparison to patients not on the drug. The mechanisms underlying this association remain under investigation, with hypotheses proposing that metformin interferes with B12 absorption by altering intestinal motility, modulating the gut microbiota, or directly binding to intrinsic factor [9].

Vitamin B12 is an essential micronutrient integral to neurocognitive and cardiovascular health [10]. Its deficiency has been linked to neurological conditions such as peripheral neuropathy (PN) and cognitive decline, both of which are prevalent among individuals with longstanding diabetes [11]. However, the causal relationship between metformin-induced B12 deficiency and these neurological manifestations remains contentious. While some studies suggest that B12 supplementation can ameliorate cognitive impairment and peripheral nerve damage, others find no significant clinical benefit [12].

Much of the existing research on this topic has been conducted in Western populations, with limited data from India despite the country's substantial diabetic burden [13]. Cross-sectional studies from India have reported associations between metformin use and decreased serum B12 levels, but longitudinal investigations exploring the subsequent impact on nerve function and cognition are scarce [14]. This gap underscores the need for region-specific, prospective studies to better understand the interplay between metformin therapy, B12 deficiency, and neurological outcomes in Indian diabetic patients.

The present study aimed to evaluate the effect of metformin therapy on serum vitamin B₁₂ levels in patients with newly diagnosed Type 2 diabetes mellitus over a six-month period. In addition, cognitive function was assessed using P300 event-related potentials recorded at Fz, Cz, and Pz electrode sites, along with functional biomarkers such as homocysteine and methylmalonic acid (MMA).

This study also explored the relationship between metformin use, vitamin B₁₂ status, and cognitive function in Indian patients with newly diagnosed T2DM. Understanding these associations may help guide clinical practice regarding the monitoring of vitamin B₁₂ levels in patients receiving metformin therapy and support strategies to prevent potential neurological complications.

## Materials and methods

Study design and participants

This hospital-based, longitudinal, observational study was conducted prospectively in the Department of Physiology at the All-India Institute of Medical Sciences (AIIMS), Bhopal, in collaboration with the Department of General Medicine. The study spanned from August 16, 2021, to August 15, 2023, and aimed to investigate the association between reduced serum vitamin B12 levels, metformin therapy in patients with type 2 diabetes mellitus (T2DM), and its impact on cognitive function.

The study received ethical clearance from the Institutional Human Ethics Committee (IHEC Order No.: LOP-2020, IM0245, dated March 15, 2020). Written informed consent was obtained from all participants following a thorough explanation of the study objectives, procedures, and confidentiality measures, using a participant information sheet provided in Hindi.

A total of 65 participants were initially recruited for the study. However, only 52 participants were included in the final analysis due to incomplete biochemical and electrophysiological data. Participants with incomplete laboratory or P300 recordings were excluded from the final dataset. Participants underwent routine clinical hearing screening prior to electrophysiological testing to exclude significant auditory impairment that could affect P300 recordings.

Inclusion criteria

Participants were adults aged 18 to 60 years, recently diagnosed with T2DM (≤3 months from diagnosis, as per American Diabetes Association (ADA) criteria), who had not yet initiated any glucose-lowering treatment and were scheduled to begin metformin at 500 mg and titrated up to 1000 mg daily as clinically indicated by the treating physician. Diagnosis was confirmed using WHO criteria: fasting blood glucose >126 mg/dL and postprandial blood glucose >200 mg/dL [15].

Exclusion criteria 

Patients were excluded if they had a history of neurological or psychiatric disorders, current use of medications affecting vitamin B12 metabolism or cognition, significant alcohol or substance abuse (defined as ≥1 drink/day for women, ≥2 drinks/day for men), pre-existing anemia (hemoglobin < 10 g/dL) or diagnosed vitamin B12 deficiency or were pregnant, a diagnosis of type 1 diabetes mellitus or pre-existing primary psychiatric, cardiac, or cerebrovascular disease, and physical or hearing impairments that would interfere with cognitive testing via P300.

Data collection and procedures

All participants underwent baseline evaluations, including fasting and postprandial blood glucose, HbA1c, serum vitamin B12, homocysteine, and methylmalonic acid (MMA). Cognitive function was assessed using P300 event-related potentials (ERPs).

P300 recording procedure

Event-related potentials (ERPs) are objective neurophysiological measures used to assess cognitive processing and detect subtle cognitive impairment. Compared with conventional psychometric tests, ERPs provide a precise temporal evaluation of neural activity associated with attention, stimulus discrimination, decision-making, and memory processes.

ERPs consist of a series of positive and negative voltage deflections generated in response to sensory or cognitive stimuli. Among these components, the P300 waveform is one of the most widely studied and clinically relevant indices of cognitive function. The P300 is a positive deflection occurring approximately 300 milliseconds after the presentation of a target stimulus and reflects processes related to attention allocation, working memory updating, and stimulus evaluation.

P300 latency represents the time required for cognitive processing and stimulus evaluation, and prolonged latency is considered an indicator of slowed cognitive processing. Conversely, shorter latency is associated with better cognitive performance. The P300 amplitude reflects the extent of neuronal resource allocation and efficiency of information processing. Reduced amplitude is generally associated with impaired cognitive function. Owing to these properties, P300 has been widely used as an objective marker for assessing cognitive dysfunction in various clinical conditions, including diabetes, neurodegenerative disorders, psychiatric illnesses, and substance use disorders.

Recording of P300 event-related potentials

In the present study, P300 ERPs were recorded for all participants after explaining the procedure and obtaining informed consent. Participants were instructed to avoid caffeine- and tannin-containing beverages for at least 10 hours prior to testing to minimize potential interference with electrophysiological recordings.

Recordings were performed in a sound-attenuated, electrically shielded, and dimly lit room using a Nihon Kohden NCV-EMG-ERP system (Tokyo, Japan). Participants were seated comfortably and allowed to relax before the procedure.

Disc-type Ag/AgCl surface electrodes were used to record scalp potentials. After appropriate skin preparation, including cleansing and gentle abrasion, electrodes were placed according to the international 10-20 system at Fz (midline frontal), Cz (midline central), and Pz (midline parietal) positions. Reference electrodes were placed on both mastoids (A1 and A2), and a ground electrode was positioned on the forehead. Conductive gel was applied to ensure stable electrical contact.

Skin-electrode impedance was maintained below 10 kΩ throughout the recording. All electrodes were connected to a junction box and monitored continuously to ensure signal quality. Recordings were obtained with participants in a relaxed state, with eyes closed and minimal movement to reduce artifacts.

The P300 waveform was elicited using a standard auditory oddball paradigm. Two types of auditory stimuli were delivered through headphones: frequent non-target tones and infrequent target (oddball) tones. Participants were instructed to identify the target stimuli by pressing a response button, ensuring active attention and cognitive engagement. Latency and amplitude of the P300 component were recorded and analyzed at each electrode site.

Follow-up assessments were conducted after six months of metformin therapy, repeating both biochemical and electrophysiological evaluations. Sociodemographic and relevant clinical data were collected using a structured questionnaire to account for potential confounding factors [16].

Biochemical assessments

Fasting venous blood samples were collected at baseline, prior to the initiation of metformin therapy, and again after six months. Serum vitamin B₁₂ levels were measured using an enzyme-linked immunosorbent assay (ELISA), with a reference range of 200-900 pg/mL. Plasma homocysteine and methylmalonic acid (MMA) levels were also estimated using ELISA methods at an accredited laboratory. Elevated homocysteine and MMA levels were considered functional indicators of vitamin B₁₂ deficiency. Glycemic control was assessed using glycated hemoglobin (HbA1c) at baseline and follow-up [17].

Sample size calculation

The sample size was calculated using G*Power software (version 3.1) for the comparison of serum vitamin B12 levels before the initiation of metformin therapy and after six months of treatment in the same patients (paired analysis). 

Based on previously published studies demonstrating a moderate reduction in vitamin B12 levels among patients receiving metformin therapy, a medium effect size (dz = 0.4) was assumed. With a two-tailed alpha error of 0.05 and a statistical power of 80%, the minimum required sample size was calculated to be 47 participants.

After accounting for an anticipated attrition rate of approximately 10%, the final sample size was determined to be 52 patients with type 2 diabetes mellitus receiving metformin therapy.

Data analysis

Data were entered into Microsoft Excel (Redmond, USA) and analyzed using IBM Corp. Released 2019. IBM SPSS Statistics for Windows, Version 25. Armonk, NY: IBM Corp. Continuous variables were assessed for normality using the Shapiro-Wilk test. As most variables demonstrated non-normal distribution, data were summarized as mean ± standard deviation and median with range where appropriate.

Comparisons between baseline and six-month post-treatment values were performed using the Wilcoxon signed-rank test for paired non-parametric data. Effect sizes (r) were calculated to determine the magnitude of change following metformin therapy and interpreted as small (0.1-0.3), moderate (0.3-0.5), and large (>0.5).

Glycemic parameters (HbA1c and random blood glucose) and biochemical markers (serum vitamin B₁₂, homocysteine, and MMA) were compared between baseline and follow-up. Correlation analyses were performed where appropriate. A p-value ≤0.05 was considered statistically significant, and all tests were two-tailed. Results were presented in tabular form using appropriate descriptive and inferential statistics.

## Results

A total of 52 newly diagnosed patients with type 2 diabetes mellitus (T2DM) were included in this hospital-based longitudinal study. The cohort predominantly consisted of males (n = 38, 73.1%), while females accounted for 26.9% (n = 14). The mean age was 46 years, indicating a largely middle-aged population. Regarding lifestyle factors, tobacco use was reported by 26.9% (n = 14) of participants and alcohol consumption by 25% (n = 13). These variables did not demonstrate a clinically significant association with the primary study outcomes. Baseline demographic and lifestyle characteristics are summarized in Table 1.

**Table 1:** Demographic and lifestyle characteristics of study participants (N = 52)

Characteristic	Values of the 52 participants
Age (years), Mean ± SD	46 ± 7.89
Male, n (%)	38 (73.1%)
Female, n (%)	14 (26.9%)
Pre-BMI (Mean ± SD)	26.90 ± 3.35
Post-BMI (Mean ± SD)	25.34 ± 2.75
Smoking/Tobacco use, n (%)	14 (26.9%)
No Smoking/Tobacco use, n (%)	38 (73.1%)
Alcohol use, n (%)	13 (25.0%)
No alcohol use, n (%)	39 (75.0%)

Glycaemic control and vitamin B₁₂ status over time

After six months of metformin monotherapy, a significant reduction was observed in HbA1c and random blood sugar levels following treatment. Median HbA1c decreased from 7.8% to 7.2% (p ≤ 0.001), and RBS decreased from 250.0 mg/dL to 185.0 mg/dL (p ≤ 0.001), both showing large effect sizes.

Vitamin B12 levels showed a significant decline in post-treatment (p ≤ 0.001). In contrast, homocysteine and methylmalonic acid (MMA) levels demonstrated a significant increase after treatment (p ≤ 0.001), with very large effect sizes. Overall, treatment resulted in improved glycemic parameters but worsening biochemical markers related to vitamin B12 deficiency (Table 2).

**Table 2:** Comparison of glycemic control and vitamin B12 levels at baseline versus 6 months after initiation of metformin therapy Statistical test used: Wilcoxon signed-rank test. Data presented as median (interquartile range). Significance level: p < 0.05 considered statistically significant.

Parameters	Pre Median (IQR)	Post Median (IQR)	Test Statistic (W)	p-value	Effect Size (r)
HbA1c (%)	7.8 (6.9–8.9)	7.2 (6.5–8.1)	112	0.001	−0.837
RBS (mg/dL)	250.0 (198.0–286.2)	185.0 (153.8–201.0)	63	0.001	−0.909
Vitamin B12 (pg/mL)	506.7 (347.3–613.5)	485.0 (332.4–592.0)	14	0.001	−0.980
Homocysteine (µmol/L)	16.5 (10.1–23.9)	17.2 (10.8–25.0)	1361.5	0.001	0.976
MMA (nmol/L)	373.6 (243.7–492.8)	392.3 (254.3–516.3)	1375	0.001	1.000

These findings support the hypothesis that although metformin therapy improves glycemic control, it may be associated with a decline in functional vitamin B₁₂ status, as evidenced by elevated homocysteine and MMA levels.

Cognitive function: P300 event-related potentials

Significant prolongation of P300 latency was observed at all electrode sites following six months of therapy (Fz, Cz, and Pz; p < 0.001). P300 amplitude showed a significant reduction at Fz and Pz (p < 0.05), while the change at Cz was not statistically significant. Detailed electrophysiological findings are shown in Table 3.

**Table 3:** Event-related potential (ERP) P300 latency and amplitude before and after treatment Data are presented as medians (interquartile range). Latency is expressed in milliseconds (ms) and amplitude in microvolts (µV). Fz (A11) represents the midline frontal region, Cz (A21) represents the midline central region, and Pz (A31) represents the midline parietal region. These are standard electroencephalogram (EEG) electrode positions corresponding to the frontal, central, and parietal areas of the brain. Statistical analysis was performed using the Wilcoxon signed-rank test, and results are reported as p-values with effect size.

Parameter	Electrodes	Pre Median (IQR)	Post Median (IQR)	Test Statistic (W)	p-value	Effect Size (r)
Latency (ms)	Fz (A11)	311.5 (280.0–349.2)	336.0 (306.0–365.2)	1164	<0.001	0.689
	Cz (A21)	314.0 (289.2–344.2)	349.0 (320.0–370.8)	1312.5	<0.001	0.905
	Pz (A31)	317.5 (278.8–342.2)	342.5 (310.0–377.0)	1250	<0.001	0.814
Amplitude (µV)	Fz (A11)	8.8 (5.0–13.9)	6.7 (3.7–13.1)	400.5	0.035	−0.346
	Cz (A21)	8.2 (4.3–12.6)	7.0 (4.2–11.8)	497	0.121	−0.250
	Pz (A31)	6.6 (4.4–9.1)	5.1 (3.2–7.0)	299.5	<0.001	−0.565

Spearman’s correlation analysis in 52 participants showed a moderate positive correlation between post-HbA1c and random blood sugar (RBS) (rho = 0.333, p = 0.016), indicating better agreement between glycemic parameters.

Serum vitamin B12 levels demonstrated a strong negative correlation with serum MMA (rho = −0.998, p < 0.001) and homocysteine (rho = −0.861, p < 0.001), suggesting that declining vitamin B12 levels were significantly associated with biochemical markers of vitamin B12 deficiency.

**Figure 1:**
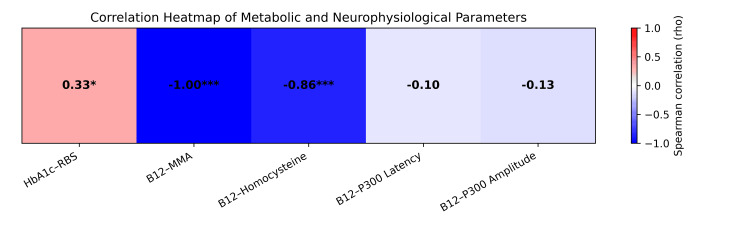
Spearman correlation between glycemic status, serum vitamin B12, metabolic biomarkers, and neurophysiological parameters (N = 52) Red: positive correlation, Blue: negative correlation, * p < 0.05: statistically significant, *** p < 0.001: highly statistically significant

No significant correlation was observed between vitamin B12 levels and P300 latency or amplitude (p > 0.05), indicating no measurable association with cognitive electrophysiological parameters within the study period (Figure 1).

Overall, metformin therapy was associated with improved glycemic control but a decline in functional vitamin B₁₂ status, accompanied by electrophysiological evidence of impaired cognitive processing.

These results suggest a deterioration in cognitive processing speed and efficiency, as indicated by increased P300 latency and reduced amplitude. This trend may reflect the emerging impact of subclinical vitamin B₁₂ deficiency, despite adequate glycemic control.

## Discussion

This prospective longitudinal study evaluated the impact of early metformin therapy on vitamin B₁₂ status and cognitive electrophysiological function in patients with newly diagnosed type 2 diabetes mellitus (T2DM). The principal findings demonstrate that, despite significant improvement in glycemic control, six months of metformin monotherapy was associated with a measurable decline in functional vitamin B₁₂ status and concurrent slowing of cognitive processing, as reflected by prolonged P300 latency.

A modest but statistically significant reduction in serum vitamin B₁₂ levels was observed following metformin initiation. Importantly, this decline was accompanied by significant elevations in homocysteine and methylmalonic acid (MMA), indicating early functional vitamin B₁₂ insufficiency even in the absence of overt biochemical deficiency. These findings are consistent with earlier studies demonstrating that metformin interferes with vitamin B₁₂ absorption, likely through impaired calcium-dependent uptake of the intrinsic factor-vitamin B₁₂ complex, altered intestinal motility, or changes in gut microbiota [18,19]. The present study extends existing evidence by highlighting that functional biomarkers may detect early vitamin B₁₂ disruption before serum levels fall below deficiency thresholds.

Cognitive electrophysiological assessment revealed significant prolongation of P300 latency across frontal, central, and parietal regions after six months of therapy. The P300 component is a well-recognized objective marker of attentional processing and working memory updating. Prolonged latency reflects delayed stimulus evaluation and reduces cognitive processing speed. The observed findings suggest early subclinical cognitive slowing in patients receiving metformin, even during short-term therapy.

These findings suggest a possible temporal association between vitamin B12 status and P300 latency; however, causality cannot be established due to the observational study design. Vitamin B₁₂ is essential for myelin synthesis and neuronal metabolism, and deficiency has been linked to impaired synaptic transmission and slowed neural conduction [20]. These mechanisms may underlie the electrophysiological changes observed in this study and suggest that emerging functional vitamin B₁₂ deficiency may contribute to cognitive alterations independently of glycemic control.

Although diabetes itself is an established risk factor for cognitive dysfunction, the longitudinal design of this study, with baseline cognitive assessment prior to metformin exposure, strengthens the temporal association between metformin therapy, evolving vitamin B₁₂ insufficiency, and cognitive slowing. This distinction is clinically important, as optimal glycemic control alone may not be sufficient to prevent early neurocognitive changes in patients with T2DM.

Current international guidelines acknowledge the association between long-term metformin use and vitamin B₁₂ deficiency. The American Diabetes Association (ADA) recommends periodic assessment of vitamin B₁₂ levels in patients receiving long-term metformin therapy, particularly in those with anemia, peripheral neuropathy, or other neurological symptoms [21]. Similarly, the UK Medicines and Healthcare products Regulatory Agency (MHRA) recognizes vitamin B₁₂ deficiency as a common adverse effect of metformin and advises testing vitamin B₁₂ levels in patients with suggestive symptoms, as well as considering periodic monitoring in individuals with risk factors such as prolonged therapy, higher doses, or pre-existing nutritional deficiencies [22]. The National Institute for Health and Care Excellence (NICE) also highlights the importance of vitamin B₁₂ monitoring in metformin-treated patients with neuropathy or anemia [23,24]. The findings suggest a possible association between vitamin B12 status and P300 latency; however, this relationship should be interpreted cautiously, as diabetes itself may influence cognitive electrophysiological responses.

This study has several strengths. It used a prospective longitudinal design, allowing evaluation of changes in vitamin B₁₂ status and cognitive function before and after initiation of metformin therapy. Cognitive assessment was performed using objective P300 event-related potentials, which provide a reliable electrophysiological measure of cognitive processing. In addition to serum vitamin B₁₂, functional biomarkers such as homocysteine and methylmalonic acid were also measured, enabling early detection of functional vitamin B₁₂ deficiency. Furthermore, the study provides valuable prospective data from an Indian population, where such evidence is limited.

However, these recommendations primarily focus on serum vitamin B₁₂ levels and symptomatic deficiency. The present study suggests that functional impairment, as reflected by elevated homocysteine and MMA levels and objective electrophysiological changes, may occur early and remain clinically silent. This finding underscores the potential need for broader monitoring strategies incorporating functional biomarkers, particularly in populations with high baseline nutritional vulnerability. The findings should be interpreted in light of certain limitations. The relatively small sample size and absence of a non-metformin or healthy control group may limit generalizability.

Dietary vitamin B₁₂ intake and baseline nutritional status were not systematically assessed. Cognitive evaluation was limited to electrophysiological measures without complementary neuropsychological testing. Additionally, the relatively short follow-up duration precludes assessment of long-term cognitive or clinical outcomes. Larger, multicenter studies with longer follow-up and interventional designs assessing the role of vitamin B₁₂ supplementation are warranted. This study has some limitations. First, no control group (such as metformin-naïve patients with type 2 diabetes or healthy individuals) was included, which limits the ability to determine causal relationships. Second, although metformin use was recorded within a clinical dose range of 500-1000 mg/day, strict dose standardization and long-term monitoring of medication adherence were not performed. Third, potential confounding factors such as dietary vitamin B12 intake and genetic variation affecting vitamin B12 metabolism were not controlled. In addition, P300 responses were analyzed at multiple electrode sites (Fz, Cz, and Pz) without formal adjustment for multiple comparisons. Future studies with larger sample sizes, appropriate control groups, and better control of confounding factors are needed to confirm these findings.

## Conclusions

In newly diagnosed Indian patients with type 2 diabetes mellitus, six months of metformin treatment led to better blood sugar control but was also linked to early signs of functional vitamin B₁₂ deficiency and a slowing of cognitive processing, shown by increased P300 latency. These findings suggest that even mild, untreated vitamin B₁₂ deficiency may affect brain function in patients taking metformin. Regular monitoring and further research of vitamin B₁₂ status, especially using functional markers such as homocysteine and MMA, should be included in routine diabetes care. Early detection and correction of vitamin B₁₂ insufficiency may help protect cognitive function and improve long-term outcomes in patients receiving metformin therapy.
